# The effect of phencyclidine-mediated blockade of NMDA receptors in the early postnatal period on glutathione and sulfur amino acid levels in the rat brain as a potential causative factor of schizophrenia-like behavior in adulthood

**DOI:** 10.1007/s43440-024-00607-3

**Published:** 2024-06-21

**Authors:** Elżbieta Lorenc-Koci, Magdalena Górny, Grażyna Chwatko, Kinga Kamińska, Małgorzata Iciek, Zofia Rogóż

**Affiliations:** 1 Maj Institute of Pharmacology, Polish Academy of Sciences, 12 Smętna Street, Kraków, 31-343 Poland; 2 The Chair of Medical Biochemistry, Jagiellonian University Medical College, 7 Kopernika Street, Kraków, 31-034 Poland; 3 Department of Environmental Chemistry, University of Łódź, 163 Pomorska Street, Łódź, 90-236 Poland

**Keywords:** Phencyclidine in early postnatal development, Glutathione deficiency, Sulfur amino acid homeostasis, Social and cognitive deficits, Neurodevelopmental model of schizophrenia

## Abstract

**Background:**

Phencyclidine, an NMDA receptor antagonist, is frequently used to model behavioral and neurochemical changes correlated with schizophrenia in laboratory animals. The present study aimed to examine the effects of repeated administration of phencyclidine during early postnatal development on the contents of glutathione and sulfur-containing amino acids, as well as the activity of antioxidant enzymes in the brain of 12-day-old rats, and schizophrenia-like symptoms in adulthood.

**Methods:**

Male Sprague-Dawley pups were administered phencyclidine (10 mg/kg) or saline subcutaneously on the postnatal days p2, p6, p9 and p12. In 12-day-old pups, 4 h after the last dose of phencyclidine, the levels of glutathione, cysteine, methionine, and homocysteine, and the enzymatic activity of superoxide dismutase (SOD), glutathione peroxidase (GPx), and glutathione reductase (GR) were measured in the frontal cortex, hippocampus, and striatum. In 70-72-day-old rats, schizophrenia-like symptoms were assessed using behavioral tests.

**Results:**

Biochemical data showed that perinatal phencyclidine treatment significantly reduced glutathione and cysteine levels in all brain structures studied, methionine was diminished in the striatum, and homocysteine in both the frontal cortex and striatum. GR activity was increased in the frontal cortex while SODactivity was decreased in the hippocampus. Behaviorally, perinatal phencyclidine induced long-term deficits in social and cognitive function and a decrease in locomotor activity assessed as the time of walking. Finally, perinatal treatment with phencyclidine resulted in a significant reduction in body weight gain over time.

**Conclusion:**

Our research provides further evidence for the usefulness of the phencyclidine-induced neurodevelopmental model of schizophrenia for studying the pathogenesis of schizophrenia.

## Introduction

Schizophrenia is a severe and complex psychiatric disease affecting ca. 1% of the general population which commonly presents in late adolescence and early adulthood. It is a heterogeneous disease with a wide spectrum of different symptoms, including positive (e.g., delusions, hallucinations) and negative (e.g., social withdrawal, anhedonia) symptoms and cognitive deficits (e.g., dysfunction of attention, working memory, and executive functions) [1]. The risk of schizophrenia development depends on genetic traits and environmental factors [2]. Although the disease exhibits high heritability [3, 4], the pattern of inheritance is non-Mendelian. The etiology of schizophrenia remains unclear, but it is commonly believed that abnormalities in the operation of genetic and environmental factors during early postnatal development initiate significant structural and functional changes in the brain [2, 5] that result in the manifestation of the typical schizophrenia symptoms in the late adolescence and/or early adulthood [6]. However, in spite of decades of intensive studies, the pathomechanism of schizophrenia still remains a mystery. On the other hand, the increasing clinical and experimental evidence suggests that oxidative stress, impaired redox regulation, N-methyl-D-aspartate (NMDA) receptor hypofunction and neuroinflammation may be key factors in the development of this disease [7–15]. Interestingly, it is strongly suggested that redox imbalance and NMDA receptor hypofunction are interlinked with each other [8, 12, 13, 16]. Some experimental data indicate that dysregulation of glutathione (GSH) biosynthesis may lie at the basis of this interconnection.

The reduced GSH is the most abundant non-protein thiol that plays a crucial role in keeping the cellular redox balance [17, 18], both through non-enzymatic reactions with various reactive oxygen species (ROS), and by acting as a cofactor of glutathione peroxidase (GPx) which catalyzes the reduction of peroxides [19, 20]. It was found that a decrease in GSH content observed in the brain of patients with schizophrenia [21–24] correlates well with the severity of negative symptoms [25]. Furthermore, in some patients with schizophrenia, a decrease in the brain GSH concentration has been shown to be associated with polymorphisms in genes encoding two subunits of γ-glutamate-cysteine ligase (GCL) [26–28], a key enzyme catalyzing the first step of GSH biosynthesis in which the dipeptide γ-glutamyl-L-cysteine (γ-GluCys) is formed from glutamate (Glu) and cysteine (Cys). The second step of GSH biosynthesis is catalyzed by glutathione synthase (GS) which ligates glycine to γ-GluCys, thus forming GSH [29, 30].

The occurrence of schizophrenia-like symptoms has also been described in rodents in which deficits in GSH content in the brain have been induced by such compounds like diethyl maleate (DEM) that causes GSH depletion [31, 32] or L-buthionine-(S, R)-sulfoximine (BSO), that blocks the enzymatic activity of GCL [14, 33–35] and using genetic manipulations in genes encoding catalytic or modifier subunits of this enzyme [36–39]. Only catalytic subunit exhibits the entire enzymatic activity and is regulated by feedback inhibition by the end product GSH [40, 41]. In addition to the activity of the catalytic subunit, the GSH level in cells depends of the availability of cysteine (Cys), an amino acid that limits the rate of GSH biosynthesis.

Regarding the relationship between the cellular GSH content and the NMDA receptor activity, recent studies have shown that blockade of these receptors by model compounds, such as phencyclidine (PCP) or MK-801, commonly used to induce schizophrenia-like symptoms in rodents, leads to inhibition of GCL enzymatic activity [16, 42]. Consistently, Baxter et al. [16] reported that the decrease in the activity of this enzyme after administration of a single dose of MK-801 to 7-day-old newborn rats was associated with a very strong reduction in the the expression of mRNA for catalytic subunit of GCL, which resulted in a distinct decline in the concentration of the synthesized GSH. In turn, Radonjič et al. [42] demonstrated that PCP administered on the postnatal days p2, p6, p9, and p12 induced long-term decreases in both mRNA expression for catalytic subunit of GCL and GSH concentration in several brain regions of 70-day-old rats. The above effects indicate that the hypofunction of NMDA receptors promotes GSH depletion in the developing brain, and that the activity of NMDA receptors is closely related to the transcriptional control of the GCL catalytic subunit. Moreover, these data suggest that GSH deficiency in the developing rat brain resulting from hypoactivity of NMDA receptors may be an important factor responsible for schizophrenia-like behavior that is manifested in adulthood. In fact, repeated administration of PCP during embryonic and early postnatal period has been shown to impair neuronal development and produce schizophrenia-like symptoms [42–46].

Our recently published studies showed that direct inhibition of GSH biosynthesis using BSO on postnatal days p5-p16 induced a small but significant decrease in GSH content only in the prefrontal cortex (PFC) and marked changes in the concentrations of the sulfur amino acids (Cys; methionine, Met; homocysteine, Hcy), especially large increases in Cys concentration, in the PFC and hippocampus (HIP) of 16-day-old rats [14]. In these brain structures, significant changes were also found in the activity of antioxidant enzymes, such as superoxide dismutase (SOD), catalase (CAT), GPx, and glutathione-disulfide reductase (GR) [47]. The consequences of all biochemical alterations in the postnatal period due to the inhibition of GSH synthesis are involved the appearance of social deficits and cognitive impairment in adulthood [14, 35]. The aim of the current study was to check the extent to which the blockade of NMDA receptors induced by repeated administration of PCP during postnatal days (p2, p6, p9, p12) affects the concentration of GSH and sulfur amino acids (Cys, Met, Hcy), as well as the activity of antioxidant enzymes (SOD, GPx, GR) in the frontal cortex (FC), HIP, and striatum (STR) of 12-day-old pups, and on the manifestation of schizophrenia-like symptoms in adulthood. The results of these studies may shed new light on disturbances in the homeostasis of thiol compounds as an important factor in the action of NMDA receptor antagonists.

## Materials and methods

All experimental procedures were carried out in accordance with the Act on the Protection of Animals Used for Scientific or Educational Purposes of January 21, 2005 reapproved on January 15, 2015 (published in Journal of Laws no. 23/2015 item 266, Poland), and in compliance with the Directive of the European Parliament and of the Council of Europe 2010/63/EU of 22 September 2010 on the protection of laboratory animals. The experimental protocols were approved by the Ethics Committee at the Maj Institute of Pharmacology, Polish Academy of Sciences, Krakow, Poland (permission no. 3/2018 of January 11, 2018). During the experiments, efforts were made to minimize the suffering of animals, and to reduce their numbers to the minimum required to obtain statistically reliable results (3R policy).

### Animals and treatment

Pregnant Sprague-Dawley females (*n* = 10) were kept in individual cages under standard housing conditions; at a room temperature (21 ± 1 °C) with 40–50% humidity and under an artificial light/dark cycle (12/12; lights on from 7 am, lights off from 7 pm). Females had unrestricted access to standard breeding feed and drinking water. On the day the Sprague-Dawley pups were born (*n* = 124), their sex was determined (58 males, 66 females), and only males were left with their dams. To maintain an appropriate number of male pups for biochemical and behavioral studies, only 56 of the 58 born were left with their mothers. In order to standardize the mother’s influence on the development of male offspring, to females with fewer males in the litter, males from other mothers were randomly added, so that the final number of males for each female was comparable (5–6 pups/mother). The phencyclidine regimen and dose were the same as in the study by Radonjić et al. [42, 48]. On postnatal day p2, p6, p9, and p12, male pups were administered subcutaneously with phencyclidine hydrochloride (PCP dissolved in saline; 10 mg/kg, *n* = 28) or saline alone (NaCl 0.9%, *n* = 28). Before the injection of each dose of PCP, newborns were weighed and the amount of PCP was adjusted to the actual body weight. No animals died during the administration of the tested substances and during adolescence.

Biochemical experiments were carried out on 4 groups of 12-day-old pups receiving PCP (*n* = 16) or saline (*n* = 16). The first set of experiments measured the concentrations of GSH and sulfur amino acids (Cys, Met, Hcy) in brain structures, such as the FC, HIP, and STR in groups of pups treated with PCP (*n* = 8) or saline (*n* = 8). The second set of experiments determined the enzymatic activities of SOD, GPx, and GR in the same brain structures of 12-day-old pups injected with PCP (*n* = 8) or saline (*n* = 8). In both experimental sets, pups were sacrificed 4 h after the last treatment.

Behavioral tests, namely social interaction test (SIT), novel object recognition (NOR) test, and open field test (OFT) assessing the expression of schizophrenia-like symptoms were performed on 2 groups of adult rats. Male pups were treated with PCP (*n* = 12) or saline (*n* = 12) in the early days after birth, weaned on postnatal day 23, and then housed in groups of four to five per cage until adulthood (p70-p74).

### Chemicals and reagents

Assay kits for the determination of enzymatic activity of SOD (Cat. No 706,002), GPx (Cat. No 703,102), and GR (Cat. No 703,202) were supplied by Cayman Chemical Company (Ann Arbor, MI, USA).

L-cysteine hydrochloride (L-Cys*HCl), glutathione (GSH), methionine (Met), homocysteine (Hcy), *N*-acetyl-cysteine (NAC), *o*-phthaldialdehyde (OPA), tris-(2-carboxyethyl)phosphine (TCEP), trichloroacetic acid (TCA), phencyclidine hydrochloride (PCP, Cat. No.: P3029) were from Sigma-Aldrich Chemical Company (Saint Louis, MO, USA).

2-Chloro-1-methylquinolinium tetrafluoroborate (CMQT) was prepared in the Department of Environmental Chemistry, University of Łódź according to the procedure described by Bald and Głowacki [49].

### Biochemical methods

#### Preparation of the tissue homogenates

The frozen tissue samples of the selected brain structures were weighed, and after thawing homogenized in 0.2 M phosphate buffer, pH 8.2 at the ratio 1: 10 (w/v, g/mL) for 0.5 min using an IKA-ULTRA-TURRAX T10 homogenizer (IKA-Werke GmbH & Co. KG, Staufen, Germany) at an operating speed of 6000 rpm. Then, the homogenates were used for biochemical assays.

### Determination of GSH and Cys content in the brain structures

Total glutathione (GSH + GSSG) and Cys concentrations in homogenates of brain structures (FC, HIP, STR) were analyzed by high-performance liquid chromatography (HPLC) with ultraviolet (UV) detection based on the method described by Bald et al. [50] which was later modified by Kamińska et al. [51].

Briefly, 100 µl of a specific brain structure homogenate (FC, HIP, STR) was incubated with 7.5 µl of 0.25 M TCEP solution at room temperature for 15 min in order to reduce disulfide bonds. In the next step, 10 µl of 0.1 M CMQT was added to the homogenate, which was then vortexed and kept at a room temperature for 5 min. The mixture was acidified with 15 µl of 3 M PCA. Then, in order to remove the precipitated proteins, the mixture was centrifuged at 12,000 rpm for 10 min at 10^o^C. Finally, 10 µl of each collected supernatant sample was injected (using an autosampler) into the HPLC system (1220 Infinity LC system from Agilent, Waldbronn, Germany) equipped with Zorbax SB-C18 column (Agilent Technologies), a diode-array detector and controlled by OpenLAB CDS ChemStation Edition software(Rev.C.01.05, Agilent Technologies, Waldbronn, Germany). Each sample was analyzed twice. The mobile phase consisted of 0.1 M TCA adjusted with 1 M NaOH to pH = 1.6 (A) and acetonitrile (B). The flow rate was set to 1 mL/min, temperature to 25^o^C and the detector wavelength to 355 nm. The following gradient elution was used to separate the 2-S-quinoline derivatives of thiols from each other and from excess reagent: 0–3.5 min, 11–25% (B); 3.5–5.5 min, 25–40% (B); 5.5–9 min 40 − 11% (B).

Identification of GSH and Cys peaks was based on the comparison of retention times and spectra from a diode array detector with the corresponding set of data obtained for standard compounds in the range of 0-300 nmol/ml for GSH and 0–30 nmol/ml for Cys. Final concentrations were expressed in nmol GSH or Cys per g of tissue.

### Determination of Met and Hcy levels

The HPLC method with fluorescence (FL) detection described previously by Borowczyk et al. [52] with some modifications was used to determine the content of Met and Hcy in brain structures. The method is based on an on-column derivatization with OPA.

Briefly, in order to reduce disulfide bonds, 14 µl of 0.25 M TCEP solution was added to 100 µl of homogenate at room temperature over 10 min. Then, 30 µl of 0.5 M NAC solution and 10 µl of 3 M PCA were added and the mixture was vortexed and centrifuged at 12,000 rpm at 10^o^C for 10 min. The collected supernatant was transferred into the HPLC system. HPLC analysis was performed on a Hewlett-Packard 1100 series system (Waldbronn, Germany) equipped with 1260 Series FL detector and controlled by HP ChemStation software (Rev. A.10.02, Hewlett-Packard Waldbronn, Germany). To perform this analysis, 5 µl of each supernatant sample was injected into the Hamilton PRP-1 column (150 nm x 4.6 nm x 5 μm). Two replicates of this analysis were performed for each supernatant sample. The mobile phase consisted of 0.01 M OPA in 0.1 M NaOH and acetonitrile in the gradient mode described earlier [52] and was applied at the flow rate 1 mL/min.

Amounts of Met and Hcy were quantified by using an FL detector at two different excitation and emission wavelengths (over 0–8 min: excitation at 348 nm and emission at 438 nm and over 8–14 min: excitation at 370 nm and emission at 480 nm). The identification of peaks was based on comparison of the retention time with data obtained for standard compounds in the range of 0–20 nmol/mL for Met and 0–7 nmol/mL for Hcy. Finally, Met and Hcy concentrations were expressed in nmoles per g tissue.

### Determination of total SOD activity

SOD activity was determined by colorimetry using SOD Cayman’s kit according to the manufacturer’s instruction. SOD activity was expressed in the conventional units U/mg of protein (1U is defined as the amount of enzyme needed for 50% dismutation of O_2_^•−^ radical). A SOD solution of known activity was used as a standard (0–0.05 U/mL).

### Determination of GPx activity

GPx activity was assayed using the GPx Cayman’s Assay Kit. The reaction of oxidized glutathione (GSSG) formation catalyzed by GPx is coupled with the GR reaction. In GR-catalyzed reaction, GSSG is reduced to GSH at the expense of nicotinamide adenine dinucleotide phosphate (NADPH) oxidation. A decrease in absorbance at 340 nm, which can be easily quantified spectrophotometrically is due to oxidation of NADPH to NADP^+^.

The measure of GPx activity in the tested samples is defined as the difference in the rate of absorbance change (∆A_340_/min) measured for the sample containing the enzyme, and in the rate of decrease in absorbance (∆A_340_/min) for the control sample.

### Determination of GR activity

Determination of GR activity was carried out using the GR Cayman’s Assay Kit. GR catalyzes the reduction of GSSG to its reduced form GSH, which is accompanied by NADPH oxidation to NADP^+^. The oxidation of NADPH causes a decrease in absorbance at 340 nm, and is directly proportional to the GR activity in the sample.

Both GPx and GR activities were expressed in nmol/mg protein/min (nmol of NADPH oxidized to NADP^+^ by the enzyme during 1 min per mg of protein).

### Determination of protein content

Protein content was assayed by the method described by Lowry et al. [53].

#### Behavioral methods

The behavioral tests used: Social Interaction Test (SIT), Novel Object Recognition (NOR) test, and Open Field Test (OFT) have been described in our previous articles [14, 35]. They were performed on the same groups of PCP or saline treated rats by experienced experimenters on consecutive days of adulthood (p70, p72 and p73).

#### SIT

Each measurement of social behavior between two rats was carried out during the light phase of the light/dark cycle. For this purpose, rats selected from separate housing cages were placed in a black PVC box measuring 67 cm × 57 cm × 30 cm (length × width × height). The arena was dimly illuminated with an indirect light of 18 lx. The difference in individual body weight between rats of a given pair was within 15 g. Rats of each pair were placed diagonally in opposite corners of the box. Social interactions between two rats were measured over a 10-min period as the total time spent in social behaviors, such as sniffing, genital investigation, chasing and fighting with each other. Every rat was used only once in the SIT. The number of social interactions was counted as a separate parameter. After each measurement session, the test box was cleaned with a 10% ethyl alcohol solution. SIT was conducted on day p70, and each group was represented by 12 rats (6 pairs).

#### NOR

After completing the SIT, on the postnatal day 71 the habituation procedure for the NOR test began. The rat was placed in an empty PVC box with dimensions: length 67 cm x width 57 cm x height 30 cm in which the floor surface was divided into 6 symmetrical sectors for 10 min to adapt it to the environment. The next day (p72), the rat was placed again in this box containing two identical objects (white tins 5 cm wide and 14 cm high) for 5 min (T1 session). One hour after T1 session, each rat was placed in the same box for 5 min (T2 session), but the box contained two different objects, i.e., the object known from the previous session, and the other new one. In both T1 and T2 sessions, for each of the two objects, the time of its exploration by the tested rat was measured separately (sniffing, touching or climbing). The recognition index was calculated for each rat [(time spent exploring the novel object– time spent exploring the familiar object)/(total time spent exploring both objects during the recognition session), and was expressed in percentages. In the NOR test, mobility was an additional parameter measured, assessed as the number of sector line crossings.

#### OFT

The elevated OFT is usually used to assess a rat’s exploratory activity in the open space. Briefly, a black circular platform without walls with a diameter of 1 m was divided into six symmetrical sectors and was raised 50 cm above the floor. The experimental room was dark and only the center of the platform was illuminated by a 75 W bulb placed 75 cm above it. In the first phase of the experiment, a rat was placed in the center of the platform and allowed to explore it. The exploratory behavior ambulation, peeping, and rearing in the open field were assessed for 5 min, by measuring, respectively, the time of walking, the number of sector crossings, and the number of episodes of peeping under the edge of the arena and rearing. The OFT was performed on day p73. Each group consisted of 12 rats.

### Statistics

The significance of differences in the exploration of the two objects by rats in the NOR test (session T1 and T2) was assessed using a two-way analysis of variance (ANOVA) followed, by the Tukey test for post hoc comparisons, when appropriate. Statistical analysis of biochemical and other behavioral data was performed using Student’s *t-test* for independent samples.

The significance of differences in the body weight between the studied groups was evaluated using the repeated measures ANOVA followed by the Newman-Keuls test for post hoc comparisons, when appropriate.

A *p* values of 0.05 or less were considered to indicate statistical significance. The statistical analysis was done using STATISTICA 10.0 Software (Statsoft, Inc, USA).

## Results

### The effect of repeated PCP administration on the contents of GSH and sulfur amino acids in brain structures

**Fig. 1:**
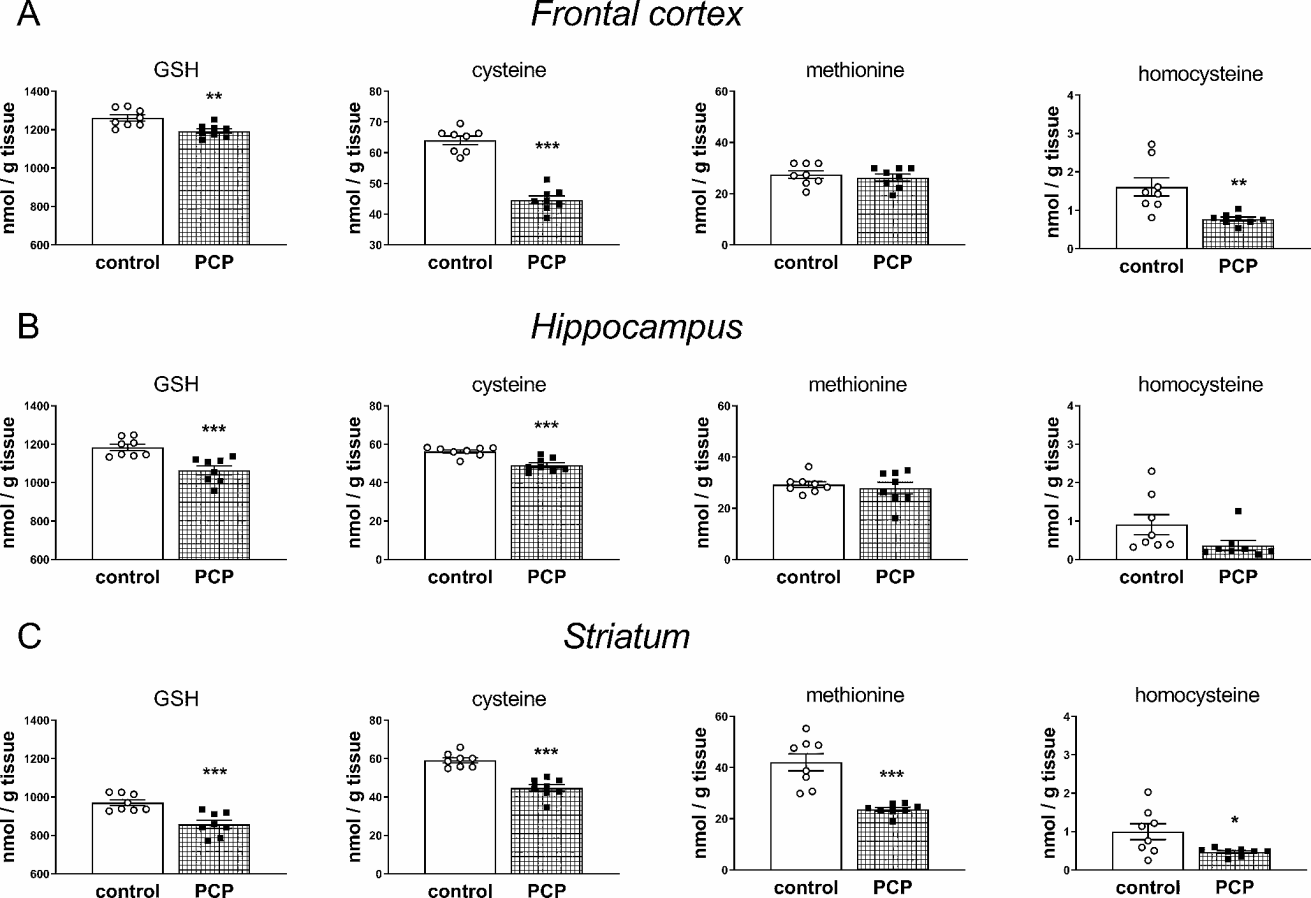
The effects of repeated PCP administration in the early postnatal days (p2, p6, p9, p12) on the concentrations of GSH, Cys, Met, and Hcy in the frontal cortex (**A**), hippocampus (**B**), and striatum (**C**) of 12-day-old pups. Data in nmole/g of tissue are presented as the mean ± SEM, *n* = 8 for each group. Statistical analysis was carried out with the Student’s t-test for independent samples, ***p < 0.001, **p < 0.01 and *p < 0.05 vs. the control group

Repeated PCP treatment in the early postnatal days resulted in significant decreases in the GSH concentration, measured in brain structures of 12-day-old male pups 4 h after the last dose, i.e. in the FC (t_14_ = 3.383, *p* < 0.01), HIP (t_14_ = 4.173, *p* < 0.001), and STR (t_14_ = 4.265, *p* < 0.001) compared to the control group (Fig. 1A–C).

Parallel to the decreases in the GSH level, the content of Cys, a rate-limiting factor in the GSH synthesis was significantly decreased in all examined brain structures (for the FC t_14_ = 10.248, *p* < 0.001; for the HIP t_14_ = 4.628, *p* < 0.001; for the STR t_14_ = 6.486, *p* < 0.001). In the PCP-treated groups, the Met concentration in the FC and HIP was at a similar level as in the control group, and only in the STR, it was significantly reduced (t_14_ = 5.345, *p* < 0.001). Hcy, which is a Met metabolite, was significantly decreased in the FC (t_14_ = 3.470, *p* < 0.01) and STR (t_14_ = 2.518, *p* < 0.05) compared to the control group, while in the HIP, only a downward trend in its content was observed (Fig. 1).

### The effect of repeated PCP administration on the enzymatic activities of SOD, GPx, and GR in brain structures

**Fig. 2:**
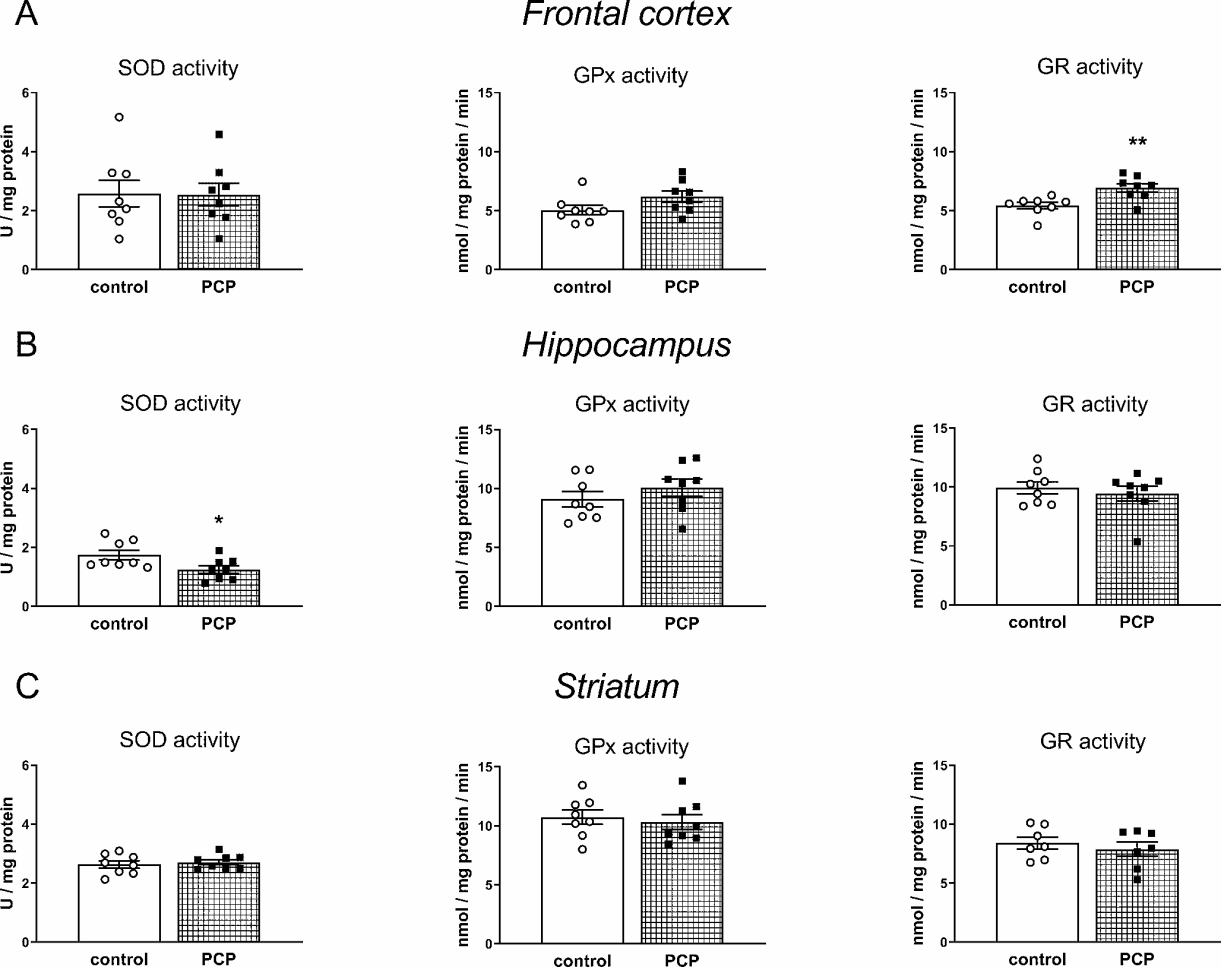
The effects of repeated PCP administration in the early postnatal days (p2, p6, p9, p12) on the enzymatic activities of superoxide dismutase (SOD), glutathione peroxidase (GPx), and glutathione-disulfide reductase (GR) in the frontal cortex (**A**), hippocampus (**B**), and striatum (**C**) of 12-day-old pups. SOD activity is expressed in the conventional unit U/mg of protein while GPx and GR activities are expressed in nmol/mg of protein/min. Further explanations are provided in the Material and Methods section. Data are shown as the mean ± SEM, *n* = 7–8 for each group. Statistical analysis was carried out with the Student’s t-test for independent samples, **p < 0.01 and *p < 0.05 vs. the control group

The activity of antioxidant enzymes (SOD, GPx, GR) in the FC, HIP, and STR of 12-day-old Sprague–Dawley male pups, was measured in tissue homogenates, 4 h after administration of the last dose of PCP or vehicle (0.9% NaCl)(Figs. 2A–C).

Of the antioxidant enzymes studied, PCP treatment affected only SOD activity in the HIP and GR activity in the FC. In particular, SOD activity in the HIP was significantly reduced (t_14_ = 2.377, *p* < 0.05), while GR activity in the FC was significantly increased (t_14_ = -3.399, *p* < 0.01) vs. the appropriate control group.

### Repeated PCP administration in the early postnatal period impairs social behavior in adulthood

**Fig. 3:**
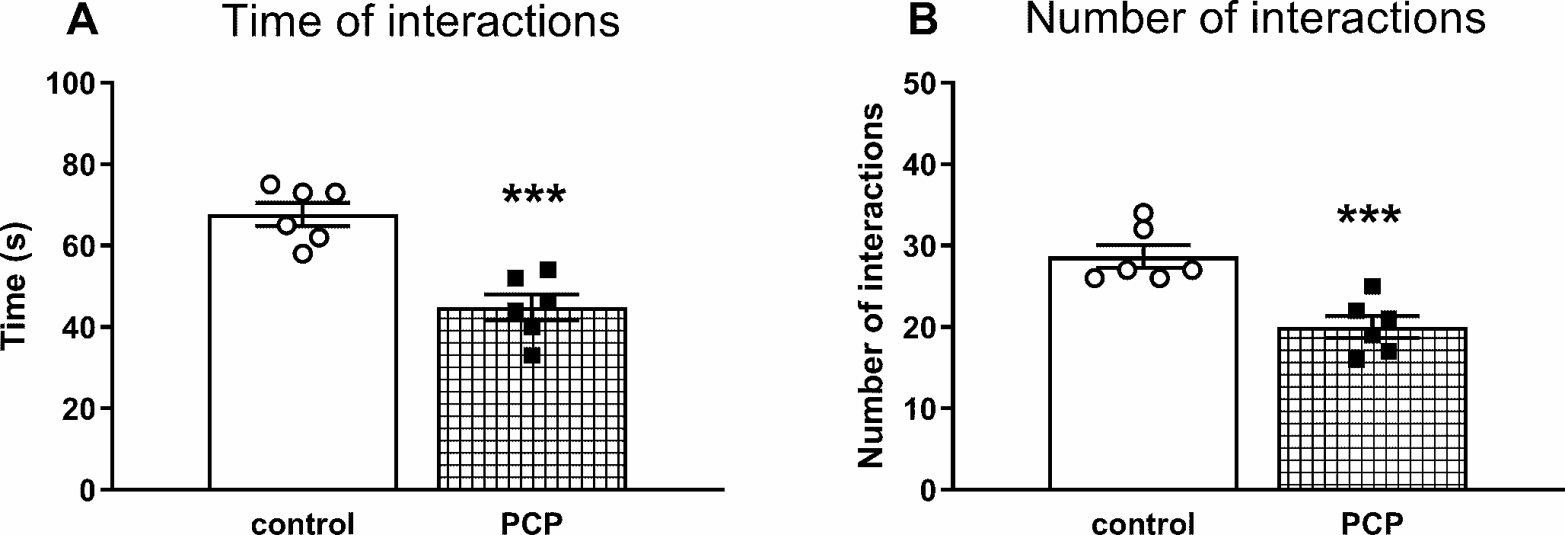
The effects of repeated PCP treatment in the early postnatal days (p2, p6, p9, p12) on social behavior deficits assessed in the SIT in 70-day-old rats as the total social interaction time expressed in seconds (**A**) and the number of interactions (**B**). Data are presented as the mean ± SEM, *n* = 12 (6 pairs) for each group. Statistical analysis was carried out with the Student’s t-test for independent samples, ***p < 0.001 vs. the control group

Statistical analysis of social behavior in 70-day-old rats revealed that repeated administration of PCP in the early postnatal period shortened the total time spent by two rats in social interactions (t_10_ = 5.361, *p* < 0.001) and reduced the number of these interactions (t_10_ = 4.420, *p* < 0.001) in adulthood (Fig. 3A and B).

### Repeated PCP administration in the early postnatal period impairs cognitive performance in adulthood

**Fig. 4:**
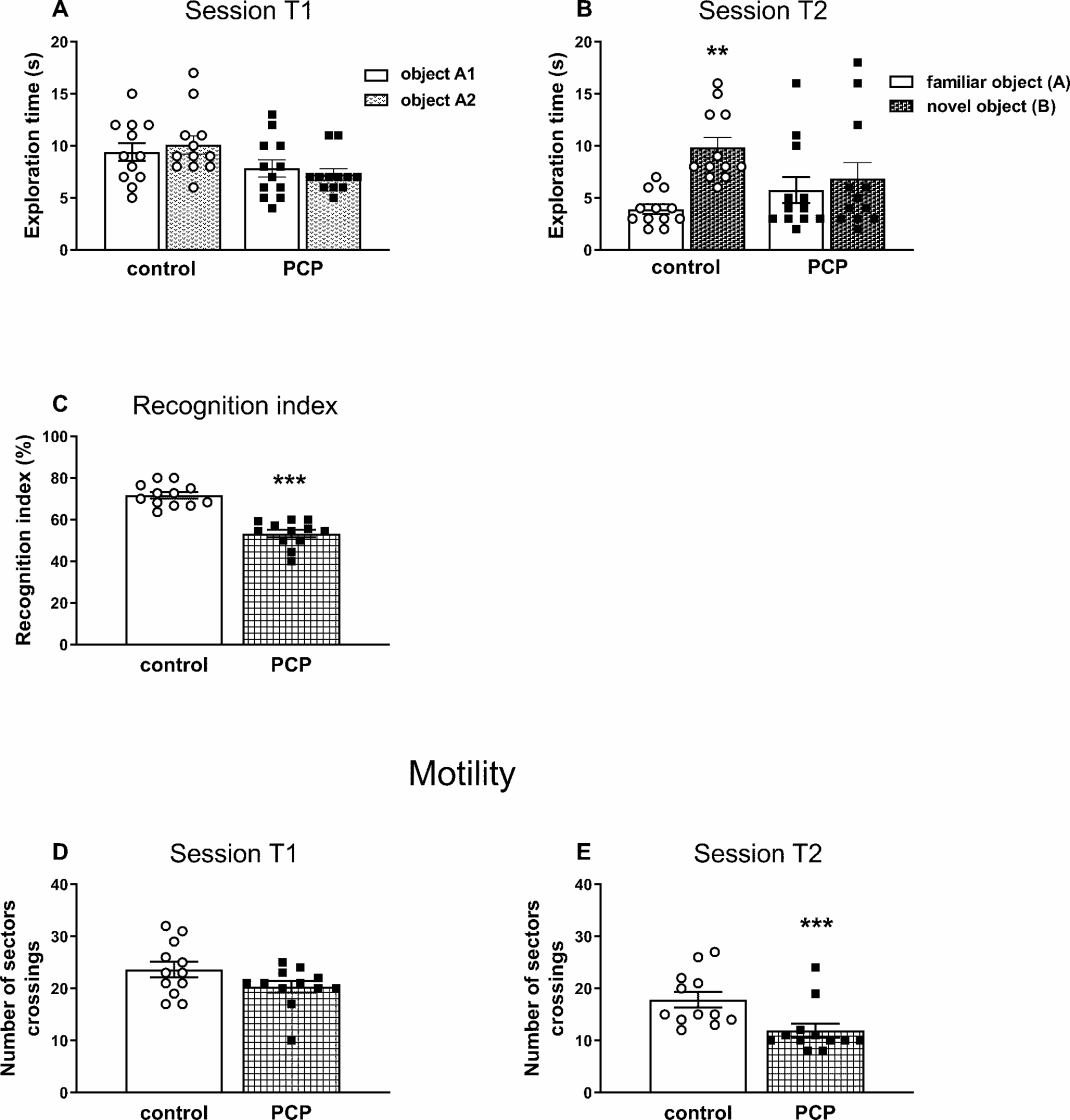
The effects of repeated (PCP administration in the early postnatal days (p2, p6, p9, p12) on cognitive performance assessed in the NOR test in adult rats. The effects of PCP on: (**A**) exploration of two objects in the T1 session, (**B**) exploration of a novel and familiar object in the T2 session, (**C**) the recognition index, and on (**D**) motility of rats in T1 and T2 sessions. Data are presented as the mean ± SEM, *n* = 12 for each group. Statistical analysis for data presented in figures A and B was performed using two-way ANOVA; asterisks indicate the significance of difference according to the Tukey post hoc test, **p < 0.01 vs. familiar object (**A**). Statistical analysis for data presented in figures **C**, **D** and **E** was carried out with the Student’s t-test for independent samples, ***p < 0.001 vs. the control group

The NOR test designed to assess cognitive impairment was performed in 72-day-old rats on the next day after SIT (Fig. 4).

A two-way ANOVA performed for the exploration time of two identical objects by each rat from PCP- or saline-treated groups during the acquisition trial (T1 session) showed a significant effect of the treatment (F_1,44_ = 7.669, *p* < 0.01), no effect of the object (F_1,44_ = 0.0027, *p* > 0.05), and no interaction between treatment and objects (F_1,44_ = 0.614, *p* > 0.05). In the T1 session both control and PCP-treated rats spent the same amount of time on exploration of two identical objects (Fig. 4A). The same analysis performed for the exploration time of two different objects (familiar and novel) by each rat from PCP or saline treated groups during the retention trial (session T2) showed no treatment effect (F_1,44_ = 0.263, *p* > 0.05), but a significant effect of the object (F_1,44_ = 4.505, *p* < 0.05), and an interaction between the treatment and object (F_1,44_ = 9.449, *p* < 0.01). Post hoc analysis showed that in the session T2, control rats explored the novel object significantly longer than the familiar one, in contrast to the PCP-treated rats which explored both objects with the same intensity (Fig. 4B).

As for the calculated value of the recognition index in the group of rats receiving PCP, it was significantly lower compared to the control group (t_22_ = 7.748, *p* < 0.0001) by 25.7% (Fig. 4C).

Regarding the motility of the tested rats, in the T1 session there was no significant differences between the PCP-pretreated and control rats in this respect, but in the T2 session motility of the PCP-pretreated rats was significantly lower than in the control group (t_22_ = 2.924, *p* < 0.01; Fig. 4D and E).

### Repeated PCP administration in the early postnatal period reduces the time of walking in adulthood

On postnatal day 72, control rats and those exposed to PCP in the early postnatal period were tested for exploratory behavior (time of walking, ambulation, peeping and rearing) in the OFT over 5-minute sessions.

**Fig. 5:**
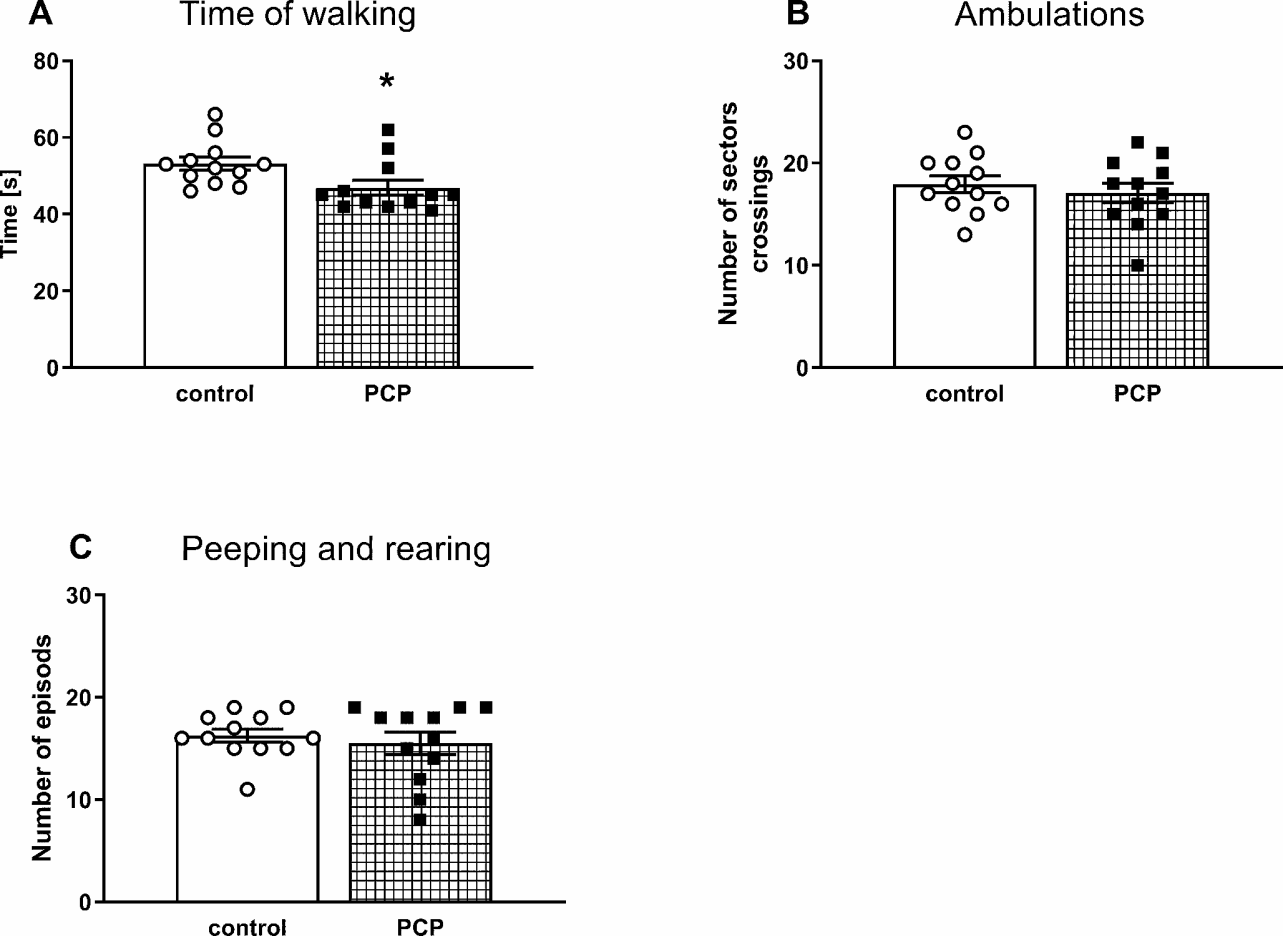
The effects of repeated PCP administration in the early postnatal days (p2, p6, p9, p12) on exploratory behavior evaluated in the OFT in adult rats as (**A**) the time of walking expressed in seconds, (**B**) the number of sector crossings, (**C**) the number of peeping and rearing episodes. Data is shown as the mean ± SEM, *n* = 12 for each group. Statistical analysis was carried out with the Student’s t-test for independent samples, ^*^p < 0.05 vs. the control group

Among the parameters studied, only the time of walking was affected by PCP, namely, it was significantly reduced in the PCP-pretreated group vs. the control group (t_22_ = 2.442, *p* < 0.05; Fig. 5A).

### The effect of repeated PCP administration in the early postnatal period on body weight of Sprague-Dawley rats

The repeated measures ANOVA performed for body weight of rats treated with saline (control) or PCP (10 mg/kg sc) during postnatal days p2, p6, p9, and p12 revealed significant effects of treatment (F_1,22_ = 21.240, *p* < 0.001) and time (F_5,110_ = 3125.28, *p* < 0.0001), and the interaction of time x treatment (F_5,110_ = 8.7899, *p* < 0.0001). Post hoc comparison of the examined groups showed significantly reduced weight gain in rats receiving PCP compared with the controls, and this effect was particularly pronounced in the late adolescence and early adulthood (p60 and p70) (Table 1).

**Table 1:** The effect of repeated PCP (10 mg/kg s.c.) administration in the early postnatal days (p2, p6, p9, p12) on weight gain during development and at two time points in late adolescence (p60) and adulthood (p70)

Postnatal day	Body weight [g]	
	Control (0.9% NaCl )	PCP (10 mg/kg)
2	8.6 ± 0.3	8.8 ± 0.3
6	18.9 ± 0.3	16.5 ± 0.4
9	28.1 ± 0.3	23.6 ± 0.6
12	38.1 ± 0.5	29.0 ± 0.9
60	308.2 ± 9.1	273.3 ± 5.0***
70	374.1 ± 8.7	337.5 ± 5.8***

Data are presented as the mean ± SEM, *n* = 12 for each group. Statistical analysis was performed using the repeated measures ANOVA. Symbols indicate significance of differences between the tested groups according to the Newman-Keuls post hoc test, ^***^p < 0.001 vs. the control, saline-treated group

## Discussion

The biochemical results obtained in our study showed that repeated injections of PCP at a dose of 10 mg/kg to Sprague-Dawley pups on postnatal days p2, p6, p9, p12, according to the schedule previously described by Radonjić et al. [42, 48], significantly decreased the contents of both GSH and sulfur amino acid Cys in the FC, HIP, and STR of 12-day-old rats. i.e. in the brain structures involved in the manifestation of schizophrenia-like behaviors in adulthood. Interestingly, the decreases in GSH concentration in these structures amounting to 5.4% in the FC, 10.1% in the HIP, and 11.6% in the STR were much smaller than the decreases in Cys concentrations in these structures: by 30.2% in the FC, by 12.6% in the HIP, and by 24.2% in the STR, although Cys availability is a primary factor limiting GSH synthesis [29, 54]. Compared to the PCP-mediated blockade of NMDA receptor, direct inhibition of the GSH-synthesizing enzyme (GCL) by BSO resulted in only a slight 7% decrease in GSH concentration in the PFC of 16-day-old rats, whereas Cys levels increased significantly in the PFC by 47.5% and in the HIP by 34.9% with no changes in the STR [14, 47]. This comparative analysis shows that in these two models of schizophrenia, a different way of modulating GSH synthesis in the early days of postnatal life leads to opposite changes in Cys concentrations in specific brain structures. These results seem to suggest potential differences in further pathways of metabolism of this amino acid.

In general, Cys is a semi-essential proteinogenic amino acid supplied in food and also produced endogenously via the transsulfuration pathway from homocysteine (Hcy), which is formed during the metabolism of Met [30, 55, 56]. Cys, in addition to being a substrate for GSH synthesis, is also a substrate for the endogenous production of hydrogen sulfide (H_2_S), which is an important regulator of the redox state and has cytoprotective and neuromodulatory properties [57, 58]. Physiological concentrations of H_2_S and its metabolites enhance the function of NMDA receptors and facilitate the induction of long-term potentiation (LTP) in the HIP [57, 59]. In patients with schizophrenia, both decreases [60] and increases [61, 62] in H_2_S concentration were found, which correlated well with the manifestation of positive and negative symptoms assessed according to appropriate diagnostic scales [60, 63]. However, the effect of H_2_S on the expression of pathological changes is unclear.

In the PCP-induced model of schizophrenia presented in this study, the level of H_2_S was not determined in the examined brain structures. Therefore, we can only speculate that the decreases in Cys content, on the one hand, may be a factor limiting production of H_2_S, and on the other, they may result from the use of Cys for the production of H_2_S. The latter assumption seems more likely. Assuming this alternative, the further action of H_2_S by adding sulfur to Cys residues at the redox site of the NMDA receptor [64, 65] can be considered as a mechanism attempting to overcome the lack of stimulation of this receptor by glutamate due to the inhibition of its activity by PCP. Unlike the PCP model, in the model induced by the BSO + GBR 12,909 combination, in 16-day-old rats, despite the high concentration of Cys, a significant decrease in H_2_S synthesis was observed in the HIP, while in adulthood, in this brain structure, an increase in the concentration of H_2_S metabolite, i.e. bound sulfane sulfur was found, while the level of free H_2_S remained the same as in the control [66]. This increase in the concentration of bound sulfane sulfur in the HIP of adult BSO + GBR 12,909 model rats coincided with an intensification of locomotor activity, while its attenuation after administration of N-acetylcysteine coincided with a decrease in the level of bound sulfane sulfur [66]. The above data indicate an important role of anaerobic Cys metabolites in the expression of some symptoms of schizophrenia and their modulation in the therapy.

On the other hand, it is also worth mentioning that Cys is an essential amino acid for the proteins synthesis, correct folding and stabilization of native, physiologically active conformations [67]. Therefore, it can be assumed that significant reduction in the Cys content in the brain structures of rats receiving PCP in the early postnatal period, impairing the synthesis of various proteins, may lead to structural and functional abnormalities. Perhaps the reduction in weight gain over time, observed in PCP-treated rats in our study, is a consequence of Cys deficiency in the early postnatal period.

In addition to lowering the Cys levels in the examined brain structures of 12-day-old pups, PCP treatment also reduced the levels of Met in the STR, and Hcy in the FC and STR. Met, like Cys, is a sulfur amino acid supplied by the diet and also endogenously synthesized via the re-methylation route from Hcy [68, 69]. The first metabolite formed during the conversion of Met to Hcy is S-adenosylmethionine (SAM) [55], which serves as the principal donor of methyl groups for different methyltransferases [70, 71], including catechol-O-methyltransferase (COMT) that metabolizes the extracellular DA to 3-methoxytyramine (3-MT). Chronic PCP treatment induces excessive release of DA in the STR [72], but significantly reduces it in the PFC [73]. Hence, in our study, the decrease in the level of Met only in the STR, 4 h after the last dose of PCP, may be due to the use of a significant pool of this amino acid for the synthesis of SAM, which may then participate in the COMT-dependent catabolism of extracellular DA to 3-MT. In our study, a significant decrease in the Hcy level in the PFC and a downward trend in the content of this amino acid in the HIP with a simultaneous decrease in the concentration of Cys and maintaining the control level of Met in both these structures suggest a shift in the balance between the processes of Hcy transsulfuration and remethylation in favor of the remethylation reaction. The intensification of the Hcy remethylation reaction in 12-day-old rats subchronically administered PCP in the early postnatal period may mean an increase in SAM synthesis in the examined brain structures, and thus may lead to abnormal epigenetic changes, some of which may be related to the etiology of schizophrenia. Further biochemical studies are required to substantiate the proposed mechanism implicating the changes in the metabolism of sulfur amino acids Cys and Met in the pathogenesis of schizophrenia.

As for the activities of antioxidant enzymes in the brain of 12-day-old rats, it was shown that 4 h after the last dose of PCP, the activity of SOD in the HIP was significantly reduced, while the activity of GR in the FC increased significantly, but no significant changes in GPx activity were observed in any of the examined brain structures.

At the behavioral level, the results of the present study showed that repeated subcutaneous injections of PCP at a dose of 10 mg/kg to Sprague-Dawley rats during early postnatal days resulted in long-term social and cognitive deficits of schizophrenic type manifested in adulthood as assessed in the SIT and NOR test, respectively. The emergence of social and cognitive deficits in this neurodevelopmental model of schizophrenia is consistent with the effects shown in previous studies where PCP administered mainly to adult rodents, either acutely or repeatedly, induced these behavioral changes [73–77]. In addition to social and cognitive deficits, PCP administration to adult rats also induced hyperlocomotion [72, 78–80], which is considered as the equivalent of positive symptoms in humans. Moreover, PCP-pretreated adult rats exhibited exaggerated hyperlocomotion compared to control in response to amphetamine [72, 79]. However, in our study, administration of PCP to male pups in the early postnatal period did not increase locomotor activity in adulthood, as measured in the OFT as the time of walking. On the contrary, the walking time was significantly decreased suggesting a decrease in locomotor activity. On the other hand, Wang et al. [43] showed that PCP administration at a single 2 mg/kg dose to adolescent 42-day-old rats, that had been repeatedly pre-treated with a high PCP dose of 10 mg/kg on the postnatal days p7, p9, p11, increased the locomotor activity to a much greater extent than in control rats receiving saline. The effect described above suggests that perinatal administration of PCP sensitized the locomotor response to PCP challenge, similarly to chronic PCP treatment in adult rats, as previously shown [80–83]. However, our study did not test the effect of a PCP challenge dose on locomotor activity in adulthood. Therefore, it can be assumed that also in the model of schizophrenia used in our study, the administration of a challenge dose of PCP to adult rats might result in an increase in locomotor activity.

In conclusion, the results of the present study showed that blockade of NMDA receptors in the early postnatal life induced by subchronic administration of a high dose of PCP (10 mg/kg) led at the biochemical level to a decrease in the concentrations of GSH and essential sulfur amino acid Cys in the FC, HIP, and STR of 12-day-old rats and at the behavioral level to the emergence of social and cognitive deficits in adulthood.

### Limitations of the study

One of the limitation factors of this work is the lack of a prepulse inhibition test. However, literature data show that PCP administered at a dose of 10 mg/kg in the same regimen as in the current work, induces sensorimotor gating deficits in rats [44]. Furthermore, PCP given in a single dose has been documented to produce transient schizophrenia-like psychoses in healthy volunteers, including positive and negative symptoms and cognitive dysfunctions [84, 85], and to exacerbate existing symptoms in schizophrenic patients [86]. The long-term use of PCP causes neuropsychological deficits that persist for several weeks. Moreover, PCP-induced psychosis is clinically difficult to distinguish from the primary psychosis of schizophrenia [85]. Therefore, PCP is commonly used as a model compound to induce schizophrenia-like behavior in basic research performed on laboratory animals. Hence, it seems reasonable to assume that the behavioral changes observed in the SIT and NOR tests in our study (despite the lack of the prepulse inhibition test) reflect changes of a schizophrenic nature, resembling the negative symptoms and cognitive deficits observed in patients with schizophrenia rather than similar changes in other psychiatric diseases (e.g. depression).

The second limiting factor is the exclusion of female rats from these studies. The rationale for this exclusion was the documented effect of estradiol on GSH synthesis. Studies by Dabrosin and Őllinger [87] indicate that 20-hour exposure of primary culture hepatocytes from Sprague-Dawley females to estradiol increased both GSH content and protein expression of GCL, the rate-limiting enzyme in GSH synthesis. Moreover, this study showed that in women, estradiol increased the concentration of GSH in subcutaneous adipose tissue in the luteal phase of the menstrual cycle. The above data suggest that in females, the presence of estradiol could interfere with the effect of NMDA receptor blockade on GSH synthesis, which we avoided by performing experiments only on males.

## Data Availability

The datasets generated during and/or analyzed during the current study are available from the corresponding author upon reasonable request.

## Abbreviations

BSO: L-buthionine-(R,S)-sulfoximine
COMT: Catechol-O-methyltransferase
Cys: Cysteine
CMQT: 2-chloro-1-methylquinolinium tetrafluoroborate
DA: Dopamine
GSH: Glutathione
FC: Frontal cortex
GLC: γ-glutamate-cysteine ligase
γ-Glu-Cys: γ-glutamate-L-cysteine
GPx: Glutathione peroxidase
GR: Glutathione reductase
Hcy: Homocysteine
Hip: Hippocampus
H_2_S: Hydrogen sulfide
Met: Methionone
3-MT: 3-methoxytyramine
NAC: N-acetyl-cysteine
NMDA: N-methyl-D-aspartate
NOR: Novel object recognition test
OFT: Open field test
OPA: O-phthaldialdehyde
PCP: Phencyclidine
PFC: Prefrontal cortex
ROS: Reactive oxygen species
SAM: S-adenosylcysteine
SIT: Social interaction test
SOD: Superoxide dismutase
STR: Striatum
TCA: Trichloroacetic acid
TCEP: Tris-(2-carboxyethyl)phosphine

## References

[1] van Os J, Kapur S, Schizophrenia. Lancet. 2009;374:635–45. 19700006 10.1016/S0140-6736(09)60995-8

[2] van Os J, Kenis G, Rutten BPF. The environment and schizophrenia. Nature. 2010;468:203–12. 21068828 10.1038/nature09563

[3] Perala J, Suvisaari J, Saarni SI, Kuoppasalmi K, Isometsa E, Pirkola S, et al. Lifetime prevalence of psychotic and bipolar I disorders in a general population. Arch Gen Psychiatry. 2007;64:19–28. 17199051 10.1001/archpsyc.64.1.19

[4] Hilker R, Helenius D, Fagerlund B, Skytthe A, Christensen K, Werge TM, et al. Heritability of schizophrenia and schizophrenia spectrum based on the nationwide Danish twin Register. Biol Psychiatry. 2018;83:492–98. 28987712 10.1016/j.biopsych.2017.08.017

[5] van Os J, Rutten BP, Poulton R. Gene-environment interactions in schizophrenia: review of epidemiological findings and future directions. Schizophr Bull. 2008;34:1066–82. 18791076 10.1093/schbul/sbn117 PMC2632485

[6] Rapoport JL, Gogtay N. Childhood onset schizophrenia: support for a progressive neurodevelopmental disorder. Int J Dev Neurosci. 2011;29:251–58. 20955775 10.1016/j.ijdevneu.2010.10.003 PMC5157162

[7] Bitanihirwe BK, Woo TU. Oxidative stress in schizophrenia: an integrated approach. Neurosci Biobehav Rev. 2011;35:878–93. 20974172 10.1016/j.neubiorev.2010.10.008 PMC3021756

[8] Do KQ, Cabungcal JH, Frank A, Steullet P, Cuenod M. Redox dysregulation, neurodevelopment, and schizophrenia. Curr Opin Neurobiol. 2009;19:220–30. 19481443 10.1016/j.conb.2009.05.001

[9] Yao JK, Keshavan MS. Antioxidants, redox signaling, and pathophysiology in schizophrenia: an integrative view. Antioxid Redox Signal. 2011;15:2011–035. 21126177 10.1089/ars.2010.3603 PMC3159108

[10] Lipton SA, Cho Y-B, Takahashi H, Zhang D, Li W, Godzik A, Bankston LA. Cysteine regulation of protein function as exemplified by NMDA–receptor modulation. Trends Neurosci. 2002;25:474–80. 12183209 10.1016/S0166-2236(02)02245-2

[11] Lorenc-Koci E. Dysregulation of glutathione synthesis in psychiatric disorders. In: Dietrich-Muszalska A, Chauhan V, Grignon S, editors. Studies on Psychiatric disorders. Oxidative stress in Applied Basic Research and Clinical Practice. 1st ed. New York, NY, USA; Heidelberg, Germany; Dordrecht, The Netherlands; London, UK: Springer; 2015. pp. 269–99.

[12] Steullet P, Cabungcal JH, Kulak A, Kraftsik R, Chen Y, Dalton TP, et al. Redox dysregulation affects the ventral but not dorsal hippocampus: impairment of parvalbumin neurons, gamma oscillations, and related behaviors. J Neurosci. 2010;30:2547–58. 20164340 10.1523/JNEUROSCI.3857-09.2010 PMC6634545

[13] Steullet P, Cabungcal JH, Monin A, Dwir D, O’Donnell P, Cuenod M, Do KQ. Redox dysregulation, neuroinflammation, and NMDA receptor hypofunction: a central hub in schizophrenia pathophysiology? Schizophr Res. 2016;176:41–51. 25000913 10.1016/j.schres.2014.06.021 PMC4282982

[14] Górny M, Wnuk A, Kamińska A, Kamińska K, Chwatko G, Bilska-Wilkosz A, et al. Glutathione deficiency and alterations in the sulfur amino acid homeostasis during early postnatal development as potential triggering factors for schizophrenia-like behavior in adult rats. Molecules. 2019;24:4253. 31766654 10.3390/molecules24234253 PMC6930621

[15] Perkins DO, Jeffries CD, Do KQ. Potential roles of redox dysregulation in the development of schizophrenia. Biol Psychiatry. 2020;88:326–36. 32560962 10.1016/j.biopsych.2020.03.016 PMC7395886

[16] Baxter PS, Bell KFS, Hasel P, Kaindl AM, Fricker M, Thomson D, et al. Synaptic NMDA receptor activity is coupled to the transcriptional control of the glutathione system. Nat Commun. 2015;6:6761. 25854456 10.1038/ncomms7761 PMC4403319

[17] Jones DP. Redefining oxidative stress. Antioxid Redox Signal. 2006;8:865–79. 10.1089/ars.2006.8.1865 16987039

[18] Jones DP. Radical-free biology of oxidative stress. Am J Physiol Cell Physiol. 2008;295:C849–68. 18684987 10.1152/ajpcell.00283.2008 PMC2575825

[19] Dringen R, Hirrlinger J. Glutathione pathways in the brain. Biol Chem. 2003;384:505–16. 12751781 10.1515/BC.2003.059

[20] Fernandez-Fernandez S, Almeida A, Bolanos JP. Antioxidant and bioenergetic coupling between neurons and astrocytes. Biochem J. 2012;443:3–11. 22417747 10.1042/BJ20111943

[21] Do KQ, Trabesinger AH, Kirsten-Krüger M, Lauer CJ, Dydak U, Hell D, et al. Schizophrenia: glutathione deficit in cerebrospinal fluid and prefrontal cortex in vivo. Eur J Neurosci. 2000;12:3721–28. 11029642 10.1046/j.1460-9568.2000.00229.x

[22] Matsuzawa D, Hashimoto K. Magnetic resonance spectroscopy study of the antioxidant defense system in schizophrenia. Antioxid Redox Signal. 2011;15:2057–65. 20712400 10.1089/ars.2010.3453

[23] Yao JK, Leonard S, Reddy R. Altered glutathione redox state in schizophrenia. Dis Markers. 2006;22:83–93. 16410648 10.1155/2006/248387 PMC3850558

[24] Gawryluk JW, Wang JF, Andreazza AC, Shao L, Young LT. Decreased levels of glutathione, the major brain antioxidant, in post-mortem prefrontal cortex from patients with psychiatric disorders. Int J Neuropsychopharmacol. 2011;14:123–30. 20633320 10.1017/S1461145710000805

[25] Matsuzawa D, Obata T, Shirayama Y, Nonaka H, Kanazawa Y, Yoshitome E, et al. Negative correlation between brain glutathione level and negative symptoms in schizophrenia: a 3T 1H-MRS study. PLoS ONE. 2008;3:e1944. 18398470 10.1371/journal.pone.0001944 PMC2275307

[26] Tosic M, Ott J, Barral S, Bovet P, Deppen P, Gheorghita F, et al. Schizophrenia and oxidative stress: glutamate cysteine ligase modifier as a susceptibility gene. Am J Hum Genet. 2006;79:586–92. 16909399 10.1086/507566 PMC1559555

[27] Gysin R, Kraftsik R, Sandell J, Bovet P, Chappuis C, Conus P, et al. Impaired glutathione synthesis in schizophrenia: convergent genetic and functional evidence. Proc Natl Acad Sci USA. 2007;104:16621–26. 17921251 10.1073/pnas.0706778104 PMC2034265

[28] Gysin R, Kraftsik R, Boulat O, Bovet P, Conus P, Comte-Krieger E, et al. Genetic dysregulation of glutathione synthesis predicts alteration of plasma thiol redox status in schizophrenia. Antioxid Redox Signal. 2011;15:2003–10. 20673128 10.1089/ars.2010.3463

[29] Dringen R, Pfeier B, Hamprecht B. Synthesis of the antioxidant glutathione in neurons: supply by astrocytes of CysGly as precursor for neuronal glutathione. J Neurosci. 1999;19:562–69. 9880576 10.1523/JNEUROSCI.19-02-00562.1999 PMC6782200

[30] Lu SC. Glutathione synthesis. Biochim Biophys Acta. 2013;1830:3143–53. 22995213 10.1016/j.bbagen.2012.09.008 PMC3549305

[31] Almaguer-Melian W, Cruz-Aguado R, Bergado JA. Synaptic plasticity is impaired in rats with a low glutathione content. Synapse. 2000;38:369–74. 11044883 10.1002/1098-2396(20001215)38:4<369::AID-SYN1>3.0.CO;2-Q

[32] Cruz-Aguado R, Almaguer-Melian W, Díaz CM, Lorigados L, Bergado J. Behavioral and biochemical effects of glutathione depletion in the rat brain. Brain Res Bull. 2001;55:327–33. 11489339 10.1016/S0361-9230(01)00484-1

[33] Castagné V, Rougemont M, Cuenod M, Do KQ. Low brain glutathione and ascorbic acid associated with dopamine uptake inhibition during rat’s development induce long-term cognitive deficit: relevance to schizophrenia. Neurobiol Dis. 2004;15:93–105. 14751774 10.1016/j.nbd.2003.09.005

[34] Cabungcal JH, Preissmann D, Delseth C, Cuénod M, Do KQ, Schenk F. Transitory glutathione deficit during brain development induces cognitive impairment in juvenile and adult rats: relevance to schizophrenia. Neurobiol Dis. 2007;26:634–45. 17459716 10.1016/j.nbd.2007.03.001

[35] Lech MA, Leśkiewicz M, Kamińska K, Rogóż Z, Lorenc-Koci E. Glutathione deficiency during early postnatal development causes schizophrenia-like symptoms and a reduction in BDNF levels in the cortex and hippocampus of adult Sprague-Dawley rats. Int J Mol Sci. 2021;22:6171. 34201038 10.3390/ijms22126171 PMC8229148

[36] Franklin CC, Backos DS, Mohar I, White CC, Forman HJ, Kavanagh TJ. Structure, function, and post-translational regulation of the catalytic and modifier subunits of glutamate cysteine ligase. Mol Aspects Med. 2009;30:86–98. 18812186 10.1016/j.mam.2008.08.009 PMC2714364

[37] Yang Y, Dieter MZ, Chen Y, Shertzer HG, Nebert DW, Dalton TP. Initial characterization of the Gclm^(-/-)^ glutamate-cysteine ligase modifier subunit knockout mouse. Novel model system for a severely compromised oxidative stress response. J Biol Chem. 2002;277:49446–52. 12384496 10.1074/jbc.M209372200

[38] Dalton TP, Dieter MZ, Yang Y, Shertzer HG, Neber DW. Knockout of the mouse glutamate cysteine ligase catalytic subunit (gclc) gene: embryonic lethal when homozygous, and proposed model for moderate glutathione deficiency when heterozygous. Biochem Biophys Res Commun. 2000;279:324–9. 11118286 10.1006/bbrc.2000.3930

[39] Dalton TP, Chen Y, Schneider SN, Nebert DW, Shertzer HG. Genetically altered mice to evaluate glutathione homeostasis in health and disease. Free Radic Biol Med. 2004;37:1511–26. 15477003 10.1016/j.freeradbiomed.2004.06.040

[40] Richman PG, Meister A. Regulation of gamma-glutamyl-cysteine synthetase by nonallosteric feedback inhibition by glutathione. J Biol Chem. 1975;250:1422–6. 1112810 10.1016/S0021-9258(19)41830-9

[41] Seelig GF, Simondsen RP, Meister A. Reversible dissociation of gamma-glutamylcysteine synthetase into two subunits. J Biol Chem. 1984;259:9345–7. 6146611 10.1016/S0021-9258(17)42703-7

[42] Radonjić NV, Knezević ID, Vilimanovich U, Kravić-Stevović T, Marina LV, Nikolić T, et al. Decreased glutathione levels and altered antioxidant defense in an animal model of schizophrenia: long-term effects of perinatal phencyclidine administration. Neuropharmacology. 2010;58:739–45. 20036264 10.1016/j.neuropharm.2009.12.009

[43] Wang C, McInnis J, Ross-Sanchez M, Shinnick-Gallagher P, Wiley JL, Johnson KM. Long-term behavioral and neurodegenerative effects of perinatal phencyclidine administration: implications for schizophrenia. Neuroscience. 2001;107:535–50. 11720778 10.1016/S0306-4522(01)00384-0

[44] Wang C, McInnis J, West JB, Bao J, Anastasio N, Guidry JA, et al. Blockade of phencyclidine-induced cortical apoptosis and deficits in prepulse inhibition by M40403, a superoxide dismutase mimetic. J Pharmacol Exp Ther. 2003;304:266–71. 12490600 10.1124/jpet.102.041798

[45] Takahashi M, Kakita A, Futamura T, Watanabe Y, Mizuno M, Sakimura K, et al. Sustained brain-derived neurotrophic factor up-regulation and sensorimotor gating abnormality induced by postnatal exposure to phencyclidine: comparison with adult treatment. J Neurochem. 2006;99:770–80. 16903871 10.1111/j.1471-4159.2006.04106.x

[46] Grayson B, Barnes SA, Markou A, Piercy C, Podda G, Neill JC. Postnatal phencyclidine (PCP) as a neurodevelopmental animal model of schizophrenia pathophysiology and symptomatology: a review. Curr Top Behav Neurosci. 2016;29:403–28. 26510740 10.1007/7854_2015_403

[47] Górny M, Bilska-Wilkosz A, Iciek M, Hereta M, Kamińska K, Kamińska A, et al. Alterations in the antioxidant enzyme activities in the neurodevelopmental rat model of schizophrenia induced by glutathione deficiency during early postnatal life. Antioxidants. 2020;9(6):538. 32575563 10.3390/antiox9060538 PMC7346228

[48] Radonjić NV, Petronijević ND, Vucković SM, Prostran MS, Nesić ZI, Todorović VR, Paunović VR. Baseline temperature in an animal model of schizophrenia: long-term effects of perinatal phencyclidine administration. Physiol Behav. 2008;93(3):437–43. 17996259 10.1016/j.physbeh.2007.10.003

[49] Bald E, Głowacki R. 2-Chloro-1-methylquinolinium tetrafluoroborate as an effective and thiol specific uv-tagging reagent for liquid chromatography. J Liq Chrom Rel Technol. 2001;24:1323–39. 10.1081/JLC-100103450

[50] Bald E, Chwatko G, Głowacki R, Kuśmierek K. Analysis of plasma thiols by high-performance liquid chromatography with ultraviolet detection. J Chromatogr. 2004;1032:109–15. 10.1016/j.chroma.2003.11.030 15065785

[51] Kamińska A, Olejarz P, Borowczyk K, Głowacki R, Chwatko G. Simultaneous determination of total homocysteine, cysteine, glutathione and N-acetylcysteine in brain homogenates by HPLC. J Sep Sci. 2018;41:3241–9. 30014601 10.1002/jssc.201800381

[52] Borowczyk K, Chwatko G, Kubalczyk P, Jakubowski H, Kubalska J, Głowacki R. Simultaneous determination of methionine and homocysteine by on-column derivatization with o-phtaldialdehyde. Talanta. 2016;161:917–24. 27769501 10.1016/j.talanta.2016.09.039

[53] Lowry OH, Rosebrough NJ, Farr AL, Randall RJ. Protein measurement with the Folin phenol reagent. J Biol Chem. 1951;193:265–75. 14907713 10.1016/S0021-9258(19)52451-6

[54] Meister A, Anderson ME, Glutathione. Annu Rev Biochem. 1983;52:711–60. 6137189 10.1146/annurev.bi.52.070183.003431

[55] Finkelstein JD. Metabolic regulatory properties of S-adenosylmethionine and S-adenosylhomocysteine. Clin Chem Lab Med. 2007;45:1694–9. 17963455 10.1515/CCLM.2007.341

[56] Vitvitsky V, Thomas M, Ghorpade A, Gendelman HE, Banerjee R. A functional transsulfuration pathway in the brain links to glutathione homeostasis. J Biol Chem. 2006;28:35785–93. 10.1074/jbc.M602799200 17005561

[57] Abe K, Kimura H. The possible role of hydrogen sulfide as an endogenous neuromodulator. J Neurosci. 1996;16:1066–71. 8558235 10.1523/JNEUROSCI.16-03-01066.1996 PMC6578817

[58] Paul BD, Snyder SH. H_2_S: a novel gasotransmitter that signals by sulfhydration. Trends Biochem Sci. 2015;40:687–700. 26439534 10.1016/j.tibs.2015.08.007 PMC4630104

[59] Kimura H. Physiological role of hydrogen sulfide and polysulfide in the central nervous system. Neurochem Int. 2013;63(5):492–7. 24036365 10.1016/j.neuint.2013.09.003

[60] Xiong JW, Wei B, Li YK, Zhan JQ, Jiang SZ, Chen HB, et al. Decreased plasma levels of gasotransmitter hydrogen sulfide in patients with schizophrenia: correlation with psychopathology and cognition. Psychopharmacology. 2018;235:2267–74. 29777287 10.1007/s00213-018-4923-7

[61] Ide M, Ohnishi T, Toyoshima M, Balan S, Maekawa M, Shimamoto-Mitsuyama C, et al. Excess hydrogen sulfide and polysulfides production underlies a schizophrenia pathophysiology. EMBO Mol Med. 2019;11:e10695. 31657521 10.15252/emmm.201910695 PMC6895609

[62] Kimura H. Hydrogen sulfide (H(2)S) and polysulfide (H(2)S(n)) signaling: the first 25 years. Biomolecules. 2021;11:896. 34208749 10.3390/biom11060896 PMC8235506

[63] Ohnishi T, Balan S, Toyoshima M, Maekawa M, Ohba H, Watanabe A, et al. Investigation of betaine as a novel psychotherapeutic for schizophrenia. EBioMedicine. 2019;45:432–46. 31255657 10.1016/j.ebiom.2019.05.062 PMC6642071

[64] Kimura Y, Mikami Y, Osumi K, Tsugane M, Oka J, Kimura H. Polysulfides are possible H_2_S-derived signaling molecules in rat brain. FASEB J. 2013;27:2451–57. 23413359 10.1096/fj.12-226415

[65] Kimura H. (2019) Signaling by hydrogen sulfide (H_2_S) and polysulfides (H_2_Sn) in the central nervous system. Neurochem Int. 2019;126, 118–25. 10.1016/j.neuint.2019.01.027 30849397

[66] Górny M, Bilska-Wilkosz A, Iciek M, Rogóż Z, Lorenc-Koci E. Treatment with aripiprazole and N-acetylcysteine affects anaerobic cysteine metabolism in the hippocampus and reverses schizophrenia-like behavior in the neurodevelopmental rat model of schizophrenia. FEBS J. 2023;290:5773–93. 37646112 10.1111/febs.16944

[67] Colovic MB, Vasic VM, Djuric DM, Krstic DZ. Sulphur-containing amino acids: protective role against free radicals and heavy metals. Curr Med Chem. 2018;25(3):324–35. 28595554 10.2174/0929867324666170609075434

[68] Finkelstein JD, Martin JJ. Methionine metabolism in mammals. Distribution of homocysteine between competing pathways. J Biol Chem. 1984;259:9508–13. 6746658 10.1016/S0021-9258(17)42728-1

[69] Finkelstein JD, Martin JJ. Methionine metabolism in mammals. Adaptation to methionine excess. J Biol Chem. 1986;261:1582–87. 3080429 10.1016/S0021-9258(17)35979-3

[70] Mandaviya PR, Stolk L, Heil SG. Homocysteine and DNA methylation: a review of animal and human literature. Mol Genet Metab. 2014;113:243–52. 25456744 10.1016/j.ymgme.2014.10.006

[71] Pries K, Gülöksüz S, Kenis G. DNA methylation in schizophrenia. Adv Exp Med Biol. 2017;978:211–36. 28523549 10.1007/978-3-319-53889-1_12

[72] Balla A, Koneru R, Smiley J, Sershen H, Javitt DC. Continuous phencyclidine treatment induces schizophrenia-like hyperreactivity of striatal dopamine release. Neuropsychopharmacology. 2001;25:157–64. 11425499 10.1016/S0893-133X(01)00230-5

[73] Cadinu D, Grayson B, Podda G, Harte MK, Doostdar N, Neill JC. NMDA receptor antagonist rodent models for cognition in schizophrenia and identification of novel drug treatments, an update. Neuropharmacology. 2018;142:41–62. 29196183 10.1016/j.neuropharm.2017.11.045

[74] Lee G, Zhou Y. NMDAR hypofunction animal models of schizophrenia. Front Mol Neurosci. 2019;12:185. 31417356 10.3389/fnmol.2019.00185 PMC6685005

[75] Mouri A, Noda Y, Enomoto T, Nabeshima T. Phencyclidine animal models of schizophrenia: approaches from abnormality of glutamatergic neurotransmission and neurodevelopment. Neurochem Int. 2007;51(2–4):173–84. 17669558 10.1016/j.neuint.2007.06.019

[76] Neill JC, Barnes S, Cook S, Grayson B, Idris NF, McLean SL, et al. Animal models of cognitive dysfunction and negative symptoms of schizophrenia: focus on NMDA receptor antagonism. Pharmacol Ther. 2010;128(3):419–32. 20705091 10.1016/j.pharmthera.2010.07.004

[77] Neill JC, Harte MK, Haddad PM, Lydall ES, Dwyer DM. Acute and chronic effects of NMDA receptor antagonists in rodents, relevance to negative symptoms of schizophrenia: a translational link to humans. Eur Neuropsychopharmacol. 2014;24(5):822–35. 24287012 10.1016/j.euroneuro.2013.09.011

[78] Kitaichi K, Yamada K, Yoneda Y, Ogita K, Hasegawa T, Furukawa H, Nabeshima T. Risperidone prevents the development of supersensitivity, but not tolerance to phencyclidine in rats treated with subacute phencyclidine. Life Sci. 1995;56:531–43. 7532775 10.1016/0024-3205(94)00482-8

[79] Balla A, Sershen H, Serra M, Koneru R, Javitt DC. Subchronic continuous phencyclidine administration potentiates amphetamine-induced frontal cortex dopamine release. Neuropsychopharmacology. 2003;28(1):34–44. 12496938 10.1038/sj.npp.1300019

[80] Jentsch JD, Taylor JR, Roth RH. Subchronic phencyclidine administration increases mesolimbic dopaminergic system responsivity and augments stress- and psychostimulant-induced hyperlocomotion. Neuropsychopharmacology. 1998;19:105–13. 9629564 10.1016/S0893-133X(98)00004-9

[81] Hanania T, Hillman GR, Johnson KM. Augmentation of locomotor activity by chronic phencyclidine is associated with an increase in striatal NMDA receptor function and an upregulation of the NR1 receptor subunit. Synapse. 1999;31:229–39. 10029241 10.1002/(SICI)1098-2396(19990301)31:3<229::AID-SYN8>3.0.CO;2-3

[82] Johnson KM, Phillips M, Wang C, Kevetter GA. Chronic phencyclidine induces behavioral sensitization and apoptotic cell death in the olfactory and piriform cortex. J Neurosci Res. 1998;52:709–22. 9669320 10.1002/(SICI)1097-4547(19980615)52:6<709::AID-JNR10>3.0.CO;2-U

[83] Xu X, Domino EF. Phencyclidine-induced behavioral sensitization. Pharmacol Biochem Behav. 1994;47:603–8. 8208780 10.1016/0091-3057(94)90165-1

[84] Javitt DC, Zukin SR. Recent advances in the phencyclidine model of schizophrenia. Am J Psychiatry. 1991;148:1301–8. 1654746 10.1176/ajp.148.10.1301

[85] Steinpreis RE. The behavioral and neurochemical effects of phencyclidine in humans and animals: some implications for modeling psychosis. Behav Brain Res. 1996;74:45–55. 8851914 10.1016/0166-4328(95)00162-X

[86] Cosgrove J, Newell TG. Recovery of neuropsychological functions during reduction in use of phencyclidine. J Clin Psychol. 1991;47:159–69. 2026771 10.1002/1097-4679(199101)47:1<159::AID-JCLP2270470125>3.0.CO;2-O

[87] Dabrosin C, Ollinger K. Variability of glutathione during the menstrual cycle-due to estrogen effects on hepatocytes? Free Radic Biol Med. 2004;36(2):145–51. 14744626 10.1016/j.freeradbiomed.2003.10.028

